# Defining an optimal cut-off point for reticulocyte hemoglobin as a marker for iron deficiency anemia: An ROC analysis

**DOI:** 10.1371/journal.pone.0288505

**Published:** 2023-07-13

**Authors:** Marah Alzu’bi, Hisham Bawa’neh, Alaa Alshorman, Jawad Alrawabdeh, Nada Odeh, Yazan Hamadneh, Mai AlAdwan, Mahmoud Odeh, Abdalla Awidi

**Affiliations:** 1 Medical School, University of Jordan, Amman, Jordan; 2 Al-Basheer Hospital, Ministry of Health, Amman, Jordan; 3 Jordan University Hospital, Amman, Jordan; 4 Cell Therapy Center, University of Jordan, Amman, Jordan; The Ohio State University, UNITED STATES

## Abstract

Reticulocyte hemoglobin (CHr) is a measure of the amount of hemoglobin in reticulocytes and a marker of cell hemoglobinization. In this study, we aimed to find the optimal cut-off point for reticulocyte hemoglobin to diagnose iron deficiency anemia using multiple methods. A total of 309 patients were included. The median age at diagnosis was 54 years. Most were females (71.2%). 68% had iron deficiency anemia. Patients with IDA had significantly lower levels of CHr compared to those who had non-IDA (p < 0.0001). The optimal cut-off value of CHr for detecting IDA, determined using various methods, was 30.15 pg. This cut-off point had a sensitivity of 87.8% and a specificity of 77.7%. CHr showed a significant positive correlation with hemoglobin, mean corpuscular volume, serum iron, serum ferritin, and transferrin saturation and a significant negative correlation with total iron-binding capacity. CHr levels correlate with most established laboratory tests for IDA. It reliably detects IDA. Our results indicate the importance of CHr in diagnosing IDA, and that CHr should be used more widely in suspected cases of IDA since it is a cheap, fast, and reliable test.

## Introduction

Iron deficiency anemia (IDA) is the most prevalent anemia globally, affecting more than two billion people worldwide. IDA may be caused by poor nutrition with insufficient dietary intake of iron or may be caused by iron loss due mostly to bleeding, or may be caused by increased demands or malabsorption. It has a spectrum of symptoms ranging from fatigue, nausea, headaches, pallor, pagophagia, geophagia, restless leg syndrome to disability. Due to the high prevalence of IDA, early screening can reduce the economic, social, and human costs of IDA [1].

Ferritin is a globular protein responsible for the intracellular storage and transport of iron [2]. Additionally, ferritin acts as a buffer against iron deficiency and iron overload [3]. Accordingly, ferritin is considered the best indicator for iron deficiency in adults [4], and serum ferritin combined with blood hemoglobin (Hb) level, soluble transferrin receptor, and transferrin saturation are the most used tests to detect IDA and iron deficiency[5].

However, using ferritin as an indicator has some drawbacks; ferritin is an acute-phase reactant, serum ferritin levels increase drastically in the presence of inflammation, giving falsely high levels even when the patient has IDA. Because ferritin acts as a buffer of iron levels in the body, low levels of ferritin only occur after the depletion of iron stores in the body. A reliable, simple and cheap test is needed to detect early IDA [6].

Reticulocytes are immature red blood cells that do not have a nucleus, however, unlike RBCs, reticulocytes still contain the remnants of their molecular machinery, a mesh-like network of ribosomal RNA [7]. Reticulocytes are the youngest erythrocytes released from the bone marrow into circulating blood, they circulate in the blood for 1–2 days before they differentiate into mature RBCs [8, 9]. Reticulocytes provide a window into the health and function of the bone marrow.

The Hb content of reticulocytes (CHr) is an attractive indicator to be used as an indicator of IDA [10], and has been investigated in previous studies.

In a 1999 study by Brugnara et al., CHr was shown to be the best diagnostic marker for iron deficiency and IDA in children [11]. In another study, CHr was a useful marker in diagnosing IDA in end-stage renal disease (ESRD) adult patients [12]. In another study, CHr was found to be an effective method to identify IDA and quickly measure the responsiveness of intravenous iron treatment in IDA patients [13].

A limiting factor for CHR is the lack of a standardized cut off point among researchers affecting the accuracy in diagnosing IDA [14]. The aim of this study is to find the optimal cut off point for CHr to diagnose iron deficiency anemia in a clinical setting using multiple methods.

## Results

### Characteristics of the sample

Of the 306 who were included, 209 (68.3%) had iron deficiency anemia, and 97 (31.7%) had anemia due to causes other than iron deficiency. There were 218 (71.2%) females. The median age at diagnosis was 54 with an IQR of 34. The median values for ferritin, hemoglobin, and CHr were 18.2, 9.2, and 27.35, respectively (Table 1).

**Table 1:** Demographic and laboratory data.

Characteristic	Frequency	Percentage
Gender		
Male	88	28.8
Female	218	71.2
Diagnosis		
Iron Deficiency Anemia	209	31.7
Anemia (Other)	97	68.3
	**Median) IQR(**	
Age	54 (34)	
Hemoglobin	9.3 (2.50)	
Ferritin	15 (210.23)	
CHr	27.30 (10.2)	
MCV	77.55 (29.0)	
WBC	6.72 (3.48)	
RDW	18.35 (4.0)	
PLT	273.00 (157.5)	
Reticulocyte Percentage	1.58(1.05)	
TIBC	360(179)	
Iron	35.85(42.32)	
TFS	10.099(21.74)	
B12	359.00(268.00)	
Folate	8.10(6.6)	
LDH	387.00(179.00)	

Patients diagnosed with iron deficiency anemia had significantly lower CHr levels compared to those diagnosed with other types of anemia (p < 0.001, mean difference = 8.41 pg).

In addition, those with iron deficiency anemia were more likely to be females compared to those with other types of anemia (p < 0.001).

### Correlation between CHr and other lab markers

Pearson’s correlation was used to check for correlations between CHr levels and other lab markers in the combined sample, among the IDA group and non-IDA group. In the combined sample, CHr was significantly associated with serum iron, TIBC, and TFS (r = .456, -.588, and .424, respectively). Amongst those with IDA alone, CHr was still found to be significantly correlated with serum iron and TIBC (r = .322, -.404, respectively). Meanwhile when testing those with non-IDA alone, the said correlation was insignificant. Table 2 shows all the different correlations between CHr and other lab markers.

**Table 2:** CHr correlation with other parameters using Pearson correlation.

Lab Marker	Correlation (Entire Sample)	P-value	Correlation (IDA Only)	P-value	Correlation (Non-IDA)	P-Value
Age	.241	< .001	.174	.036	-.026	0.807
Hemoglobin	.227	< .001	.482	< .001	-.305	.003
WBC	.075	.203	-.006	.935	-0.45	.666
MCV	.343	< .001	.796	< .001	.339	<0.001
RDW	-.005	.928	-.021	.765	.391	<0.001
PLT	-.288	< .001	-.080	.267	-.302	.003
Retics Percentage	.254	< .001	.281	< .001	.236	.022
Ferritin	.283	< .001	.241	< .001	.007	.949
Iron	.456	< .001	.322	< .001	.109	.298
TIBC	-.588	< .001	-.404	< .001	-.023	.825
TFS	.424	< .001	.133	.063	.177	.088
B12	.124	.043	.033	.653	-.155	.162
Folate	.165	.011	.240	.003	-0.94	.404
LDH	.349	< .001	.213	.016	.450	<0.001

### The diagnostic value of CHr in diagnosing iron deficiency anemia

**Fig 1:**
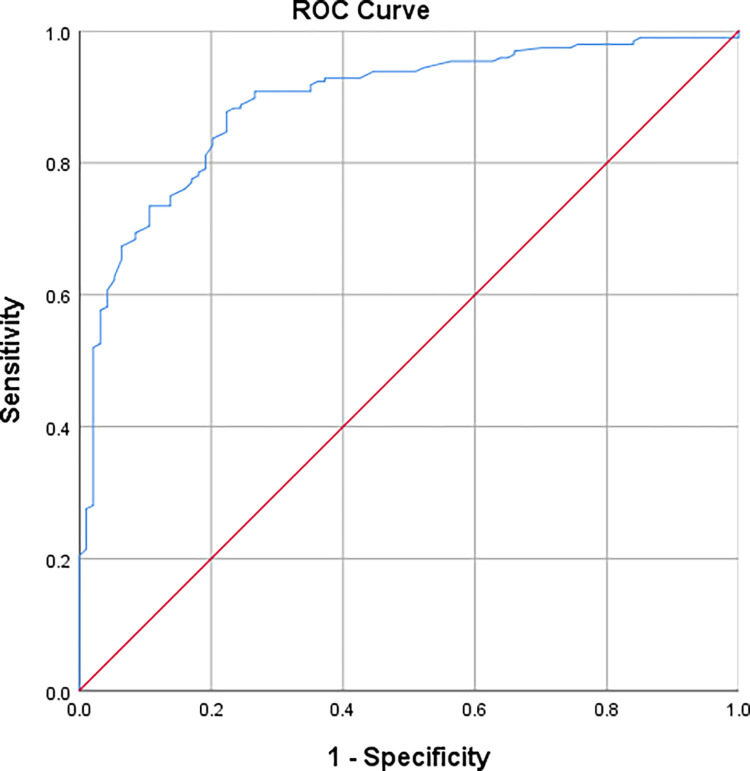
Roc curve showing the diagnostic efficacy of CHr in diagnosing IDA.

A ROC curve was used to determine the diagnostic value of CHr in diagnosing iron deficiency anemia as previously defined in the methods section. CHr showed high accuracy in diagnosing IDA (AUC = 0.891, p < 0.0001). The ROC curve is shown in Fig 1.

### Determining the optimal cut-off point for diagnosing iron deficiency anemia

The ROC curve analysis generates a table with multiple cut-off points, each with a certain specificity and sensitivity value. Using 4 different methods being: Youden index, the closest to (0,1) criteria, the concordance probability method, and the index of union. An optimal cut-off point was determined using each of these methods and compared to the others. The optimal cut-off point was 30.15 pg with a sensitivity of 0.878 and a specificity of 0.777. The point was found to be the same using any of the different methods.

## Discussion

This study looked at the value of CHr and the cutoff point to be used for IDA in adolescents and adults. Most were adults and this is reflected in the population’s median age which was 54 years with predominance of females constituting 71.2%. The female predominance is in line with medical literature [15].

CHr was found to be a useful parameter that can be confidently utilized for the diagnosis of IDA [16].Our study analyzed the CHr in IDA and non-IDA populations. We showed that patients diagnosed with IDA had significantly lower CHr levels compared to those diagnosed with non-IDA. These results are consistent with previous published work [17].

The optimal cut-off point for CHr to diagnose IDA is hotly debated. A 2022 meta-analysis found the cutoff value of CHr to be 28.2 pg with a 84% sensitivity and a 91% specificity. It is important to note that this meta-analysis has combined studies conducted with different methods. In addition, no formal methods or statistical techniques were used to define the optimal cut off point in the studies included in the meta-analysis. This leaves the question of the optimal cut off point unanswered.

The aforementioned meta-analysis showed that CHr is a more effective marker in determining IDA compared to both SF and MCV levels along with many of the other more commonly used parameters [18].

Our work indicates that CHr cutoff value of 30.15 pg can identify IDA with a 87.8% sensitivity and 77.7% specificity. The aforesaid cut-off value is similar to the 30.7 pg cut-off value reported by Auerbach et al. (2021) in a cohort of 556 patients [13]. Additionally, a number of recent studies reported similar cut-off values ranging from 30 pg to 30.9 pg [19, 20]. Nevertheless, none of these studies had a sensitivity higher than that of our study.

The uniqueness of our study lies in the fact that it defines an optimal cut-off point using multiple different statistical methods. This -to our knowledge- has not been done previously in literature. Previous studies reported wide ranges of cut-off points, and the aforementioned meta-analysis didn’t help specify any value as it included heterogenous studies that didn’t utilize any statistical methods for determination of cut-off points. We believe that our study will help fill the gap created by the wide range of cut-off points and help pave the way for future endeavors. In addition, no studies have been published on the Jordanian population specifically. Our study is the first to test this in our population.

In the present study CHr had a strong association with serum iron, ferritin, TFS, Hb, and MCV as well as it inverse relationship with TIBC. However, CHr showed no association with either WBC or RDW. This is in line with the results of a 2020 South Asian study [21].

There are two possible limitations of this study. First, the low count of males in the registry as it would have been better if the it included more males. Second, the study may not be representative of the whole population in Jordan since it was limited to one center

The size of our sample is acceptable, but larger study with more patients is warranted. It would be useful to extend the study by including patients with other comorbidities as well. Despite of these limitations, we believe that our cutoff value is satisfyingly sensitive since it remained consistent in all of the various methods we used to determine the cutoff value. It is to be noted that according to what is already published on the role of CHr in the detection of IDA, CHr is not adopted into clinical practice yet due to the lack of a universal approval on the most specific and sensitive CHr cutoff point for IDA diagnosis and this is the problem we aimed to solve in our study.

## Conclusion

CHr is a simple, cheap, fast, and reliable test for the diagnosis of IDA. The cutoff value of 30.5pg seems to be the best value that separates IDA from the rest of non-Iron deficiency anemias.

## Materials and methods

### Study population and design

A hospital-based registry of IDA in adolescents and adults with either IDA or non-IDA was analyzed retrospectively in a cross-sectional study. Data in the registry was collected from 306 patients in a university hospital. The data include age, gender, diagnosis (IDA or anemia without ID), hemoglobin, ferritin, CHr, MCV, WBC, RDW, Platelet count, reticulocytes count, serum iron, total iron binding capacity (TIBC), transferrin saturation (TFS), B12, folate and LDH. Iron deficiency was diagnosed based on either a low serum ferritin (<30 ng/mL), and/or a low TSAT (< 20%).

### IRB approval and informed consent

This study was approved by the IRB at the Jordan University hospital. No informed consent was required for this study because sample and data were de-identified and no additional blood was drawn.

### Data analysis

SPSS version 26 was used for analysis. All continuous data are presented as median with interquartile range (IQR), all categorical data are described as frequency and percentage. Quantitative variables were analyzed using Student’s t-test. Pearson’s correlation coefficient was used to assess the correlation between parameters.

Receiver operator characteristics (ROC) curve analysis was used to determine the value of CHr in identifying IDA, with results presented as the area under the curve (AUC) with corresponding 95% CI and P-value. The optimal cut-off point for CHr was determined using Youden’s index (J) and validated using closest to (0,1) criteria (ER), concordance probability method (CZ) and Index of Union (IU) [22].

## Supporting information

S1 Data (CSV)
